# Imaging of electric-field-induced domain structure in DyMnO$_{3}$ nanocrystals

**DOI:** 10.1186/s11671-024-04165-8

**Published:** 2024-12-15

**Authors:** Mansoor A. Najeeb, Robbie Morrison, Ahmed H. Mokhtar, Daniel G. Porter, Frank Lichtenberg, Alessandro Bombardi, Marcus C. Newton

**Affiliations:** 1 Department of Physics and Astronomy, University of Southampton, Southampton, SO17 1BJ UK; 2 Diamond Light Source, Harwell Oxford Campus, Didcot, OX11 0DE UK; 3 Department of Materials, ETH Zürich, Zürich, 8093 Switzerland

**Keywords:** Multiferroic, Domain walls, Bragg coherent diffraction imaging, Machine learning

## Abstract

Multiferroic materials that exhibit interacting and coexisting properties, like ferroelectricity and ferromagnetism, possess significant potential in the development of novel technologies that can be controlled through the application of external fields. They also exhibit varying regions of polarity, known as domains, with the interfaces that separate the domains referred to as domain walls. In this study, using three-dimensional (3D) bragg coherent diffractive imaging (BCDI), we investigate the dynamics of multiferroic domain walls in a single hexagonal dysprosium manganite (h-DyMnO$_3$) nanocrystal under varying applied electric field. Our analysis reveals that domain wall motion is influenced by the pinning effects, and a threshold voltage of +3 V is required to overcome them. Using circular mean analysis and phase gradient mapping, we identified localised phase realignment and high-gradient regions corresponding to domain walls, providing insights into the behaviour of multiferroic systems under external stimuli.

**Supplementary Information:**

The online version contains supplementary material available at 10.1186/s11671-024-04165-8.

## Introduction

### Multiferroics synopsis

Multiferroics are of great interest, as an understanding of the interplay between coexisting yet contrasting ferroic properties at the atomic scale that could lead to the development of novel technologies, where one ferroic property is used to control the conjugated field of another.[1–5] For example, multiferroics where ferroelectric and ferromagnetic orders are coupled, allowing the control of magnetic order through the use of an external electric fields and vice-versa.[6–10] Multiferroics also exhibit varying regions of polarity, known as domains. The interfaces that separate these domains are known as domain walls. These domain walls can be created, moved or erased with application of external stimuli. Importantly, even in the absence of external interference, the domain walls can give rise to intrinsic defects and strain within the material which results in local atomic rearrangements.[11] Domain walls in multiferroics are also 2D systems that can host functional electronic and magnetic properties, which could find utility in new generation devices due to their agility and spatial mobility.[12, 13] As a result, there is a vibrant effort to realise technologies such as integrated devices that utilise this underlying mechanisms. Multiferroics are generally classified into two types. Type-II multiferroics are those in which ferroelectricity emerges as a result of specific magnetic ordering. Type-I encompasses all others where ferroelectricity does not have a magnetic origin. Further classification is possible based on: (1) whether or not ferroelectricity is the primary order parameter; or (2) the atomic scale mechanism that gives rise to ferroelectricity, namely electronic lone pairs, geometric constraints, charge ordering or magnetic ordering with resulting inverse Dzyaloshinskii-Moryia interaction.[14, 15]

### BCDI synopsis

In multiferroic materials, an applied external electric field can alter the ordered electric dipole moments, which leads to significant changes in the material’s microstructural characteristics which manifest as domain wall movements and associated strain. The intensity of diffraction patterns can be influenced by various aspects of crystal structure including unit cell parameters, crystal defects and grain boundaries.[16] When an external voltage is applied, new defects may arise and existing defects may shift within the crystal.[17] Conventional techniques like X-ray diffraction and scanning electron microscopy, often provide average information across larger volumes, missing local variations that are crucial for understanding strain and defect dynamics. In contrast, BCDI presents a powerful alternative, as it offers high-resolution 3D imaging capabilities that enables precise tracking of changes in the crystal structure. The process involves shining a spatially coherent X-ray on to the nanoscale crystal configured at the Bragg reflection geometry. The coherence length exceeds the dimensions of the crystal [18, 19], leading to corresponding interference patterns in the far field, thus producing a comprehensive 3D $k$-space diffraction pattern and the intensity is measured by a photon counting area detector. The experiment captures a two-dimensional diffraction pattern on the detector, while the third dimension is acquired by incrementally rocking the sample and recording the diffraction pattern at each step (rocking curve scan).[20] Subsequent to this, machine learning-aided iterative phase reconstruction methodologies are employed to recover the distinct 3D electron density and phase information [21]. The displacement of ions throughout the material correlates directly with the phase, enabling the derivation of strain information via the relationship $\phi = \textbf{Q} \cdot \textbf{u}$, where $\textbf{u}$ represents atomic displacement [18, 22]

### Summary of manuscript

In this manuscript, we use BCDI reconstructed 3D images to reveal the phase variations within h-DyMnO$_3$ nanocrystals as a function of applied voltage. Although this approach offers valuable insights into the electric-field-induced domain wall structure, the direct investigation of magnetic and magneto-electric properties of multiferroic material falls outside the scope of this study.

## Results

### Bragg CDI experiment

DyMnO$_3$ nanocrystals were grown using the bulk melt-grown technique described by [23, 24] were prepared using the procedure described in the methods section. Powder XRD (SI Fig. S12) analysis at the laboratory was conducted prior to the synchrotron measurements to ensure the right hexagonal crystallographic phases were obtained with lattice parameters a = b = 6.18 Å and c = 11.4 Å [25]. The formation of ferroelectric domain structures in h-DyMnO$_3$ can be achieved by cooling the material from its high-symmetry paraelectric phase (P6$_{3}$/*mmc*) to the lower-symmetry ferroelectric phase (P6$_{3}$*cm*) at a critical temperature ($T_{c}$) exceeding 1250 K.

**Fig. 1:**
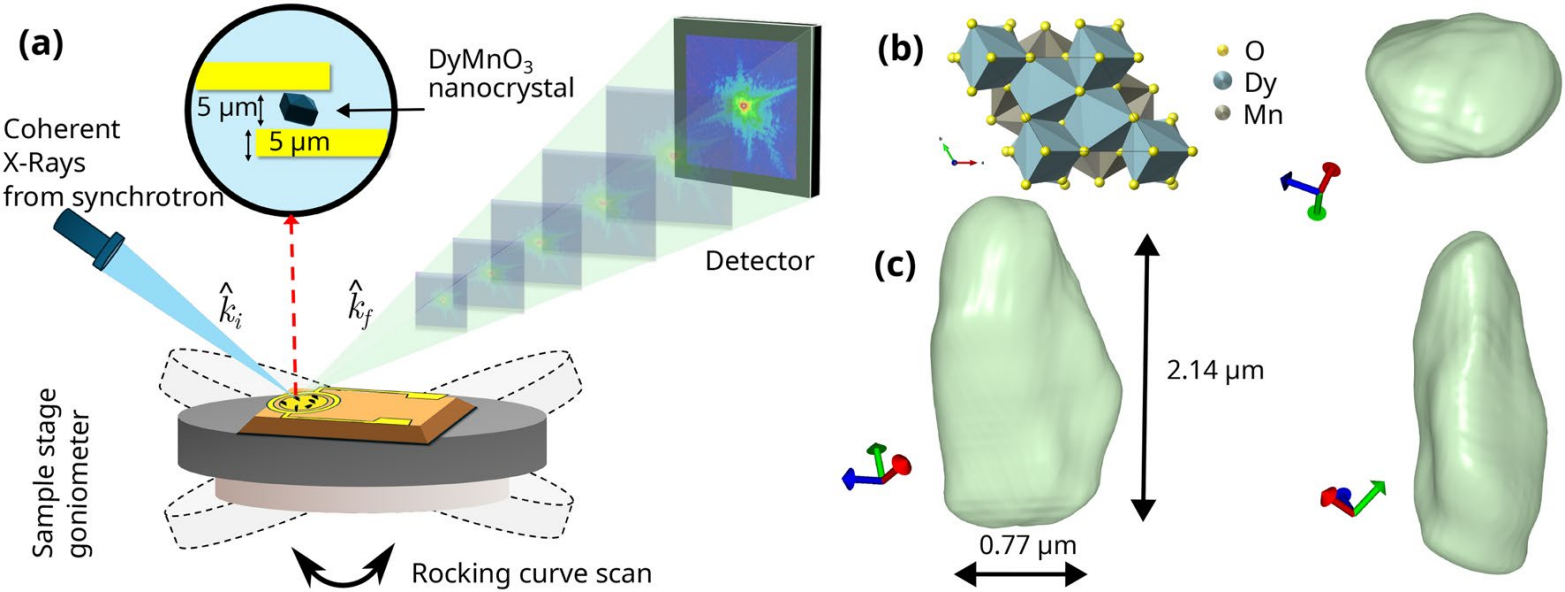
**a** Coherent Diffraction Imaging setup showing the incident (ki ) and reflected (kf ) X-ray wave vectors, inset shows the graphical representation of h-DyMnO$_3$ nanocrystal deposited on gold interdigitated electrodes (IDEs). **b** Crystal structure of h-DyMnO$_3$ (P6$_{3}$*cm*), **c** Reconstructed volume of a single h-DyMnO$_3$ nanocrystal showing three orthogonal projections at an isosurface of 97%

BCDI characterisation was performed at Beamline I16 at the Diamond Light Source (DLS). The graphical representation of the BCDI experimental setup used is given in Fig. 1(a). A channel-cut Si (111) monochromator was used to produce a beam of 9 keV X-rays in Bragg geometry. The beam was then focused onto the IDE sample mounted on the stage positioned at the eucentric point of the diffractometer. The sample was rotated to locate the (112) reflection and diffraction data were acquired using a QuadMerlin photon-counting area detector mounted in the reflection geometry. The sample was positioned 1,310 mm away from the detector. Rocking curve scans consisting of 201 steps with a step size of 0.003$^{\circ }$ and an exposure time of 5 s were performed around the peak maxima. Measurements were taken at a range of voltages from $0.0\,\text {V}$ to $\pm 5\,\text {V}$ on a single isolated h-DyMnO$_{3}$ nanocrystal.

### Machine learned phase retrieval

The initial data preparation and iterative phase retrieval process in this study utilised “Bonsu: The Interactive Phase Retrieval Suite” software package [26]. A cuboid support was created to provide geometrical constraints during the phase retrieval process. The hybrid input–output (HIO) mask algorithm was then performed, running for 2000 iterations to refine the phase information and reconstruct the image. The algorithm iteratively refines an initial guess of the object’s phase by switching between real-space and reciprocal-space representations. The algorithm iterates these steps until it converges to a stable object. Once a reasonable morphology is achieved, the initial cuboid support is further refined by rotating, cropping and Gaussian smoothing to match with the object and later used in the ML reconstruction process.

A convolutional neural network (CNN) architecture based deep learning model was employed for ML phase retrieval [27]. The network was initially trained by the propagation of 25,000 simulated diffraction patterns which were then compared with the corresponding Fourier pair ground truth objects. Training was conducted utilizing back-propagation over at least 2000 epochs and implementing the ADAM (Adaptive Moment Estimation) optimiser. Following the initial training phase, a transfer learning strategy was employed, applying the pre-trained network to experimental diffraction patterns across several hundred epochs. The optimisation during this phase was guided by the loss metric comparing the Fourier transform of the predicted object against the provided diffraction pattern. This transfer learning phase enhances the model’s robustness and provides a precise fine-tuning mechanism, making it more adaptable to specific data sets.

### BCDI of ferroelectric domain wall movements

**Fig. 2:**
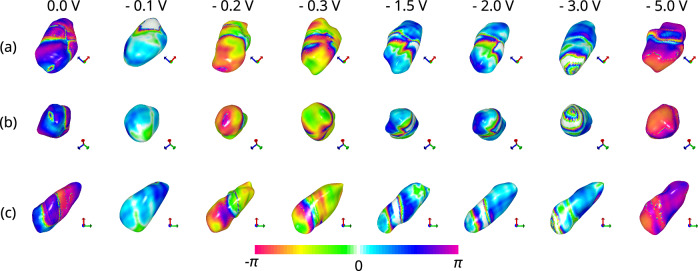
BCDI reconstruction of a single h-DyMnO$_3$ nanocrystal showing the morphological features and mapped phase information to the surface of the object at (112) reflection. Each row corresponds to the view of the crystal along the 3 orthogonal axes **a** x-axis, **b** y-axis, and **c** z-axis, and the columns from left to right show the surface and morphological changes under various applied voltages: 0.0 V, $-$0.2 V, $-$0.3 V, $-$1.5 V, $-$2.0 V, $-$3.0 V, and $-$5.0 V. The colour map shows the phase range of $-\pi$ and $\pi$ displayed at the bottom of the figure which corresponds to compressive and tensile strains respectively. While the overall morphology of the crystal remains consistent across the applied voltages, significant phase changes are visible on the surface. At 0.0 V the crystal shows a uniform phase distribution indicated by symmetrical stripe phase pattern and predominant magenta and blue colours. As the voltage reaches $-$0.5 V, the phase shifts more towards the $-\pi$ with the appearance of more orange and red colours, indicating increased compressive strain

**Fig. 3:**
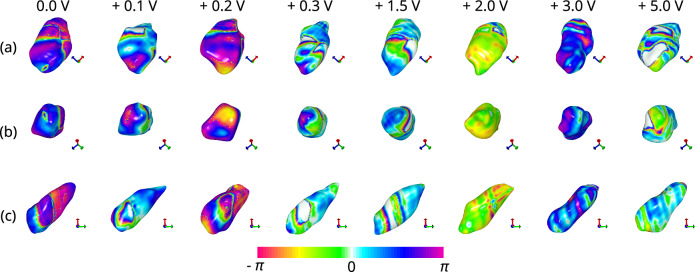
Morphology and phase mapping of h-DyMnO$_3$ nanocrystal viewed along the 3 orthogonal axes **a** x-axis, **b** y-axis, and **c** z-axis, under positive voltages (0.0 V, +0.2 V, +0.3 V, +1.5 V, +2.0 V, +3.0 V, and +5.0 V). Same images of 0.0 V from Fig. 1 is used for consistency. As the positive voltages increase, the stripe phase pattern gets distorted demonstrating the effect of the applied voltage on the surface phase distribution

Initial scans were taken without applying any voltage to the nanocrystal. Then, a voltage of $-0.2\, \text {V}$ was applied, and rocking curve scans were performed. Subsequently, the following voltages were applied in sequence: $\pm 2\, \text {V}$, $\pm 0.1\, \text {V}$, $\pm 0.3\, \text {V}$, $\pm 1.5\, \text {V}$, $\pm 3\, \text {V}$, and $\pm 5\, \text {V}$. Figure 2 and Fig. 3 shows the BCDI reconstruction of a single h-DyMnO$_3$ nanocrystal showing the morphological features and mapped phase information to the surface of the object at (112) reflection while applying -ve and +ve voltages respectively. The crystal is viewed at 97% isosurface and has an average dimension of 2.14 $\mu \text {m}$ x 1.26 $\mu \text {m}$ x 0.77 $\mu \text {m}$. The resolution for the 0.0 V single crystal was estimated using the Phase Retrieval Transfer Function (PRTF) [28] and was determined to be 15.83 nm (Fig. S10 of Supplementary Material). Across the varying voltages, the crystal morphology remains consistent showing the effectiveness of the ML reconstruction. The colour map shows the phase range of $-\pi$ and $\pi$ which corresponds to compressive and tensile strains respectively. Specifically, the colour map serves as a direct representation of the phase shifts within the crystal, facilitating an understanding of the domain configurations and their evolution under applied electric fields. At 0.0 V the crystal shows a uniform phase distribution indicated by symmetrical stripe phase pattern and predominant magenta and blue colours (towards the $\pi$ side). Figure 3 shows that, as the voltage reaches $-$0.5 V, the phase shifts more towards the $-\pi$ with the appearance of more orange and red colours, indicating increased compressive strain. Figure 3 shows the effect of +5.0 V on the crystal where the stripe phase pattern looks distorted while the strain profile remains close to the neutral area.

**Fig. 4:**
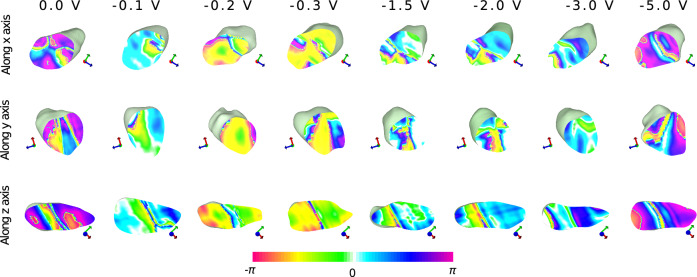
Slices of reconstructed h-DyMnO$_3$ nanocrystal under negative voltages sliced along three orthogonal axes. The symmetrical stripe phase pattern presented on the surface of the crystal is also visible in the slices of 0.0 V. As the negative voltage increases, noticeable phase variations occurs, reflecting changes in the crystal structure in response to the applied electric field

**Fig. 5:**
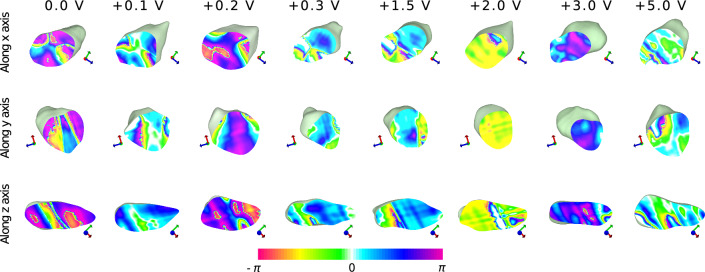
Slices of reconstructed h-DyMnO$_3$ nanocrystal under positive voltages sliced along three orthogonal axes. A clear phase variation is visible between 0.0V and +0.2 V where the cyan, white and green phases (which are close to neutral) widen and move apart while the magenta phase, which is at the maximum of $\pi$, spreads across the bulk of the crystal

#### Voltage-induced phase variations

Figure 4 and Fig. 5 shows slices through the centre of reconstructed electron density map of h-DyMnO$_3$ nanocrystal under negative and positive voltages sliced along three orthogonal axes respectively. Figure S1-S6 in the Supplementary material shows multiple slices shown at five approximately 150 nm - 250 nm intervals for each orientations. At 0 V without any external field applied, the slices show a relatively balanced mix of colours ranging from dominant magenta and blue colours (towards the $\pi$ side) along with yellow and red (towards the $-\pi$ side). The phase distribution reflects the inherent strain within the crystal due to its internal structure and any pre-existing lattice distortions. The symmetrical stripe phase pattern visible on the surface (Fig. 3) can be seen on the bulk of the crystal along the x-axis. As the negative voltage increases (Fig. 4), the phase shifts more towards the blue and green region, which correspond to negative phase values indicating compressive strain. Figure 5 shows, as the positive voltage increases, the crystal experiences more tensile strain, leading to a shift in the phase difference distribution towards positive values.

**Fig. 6:**
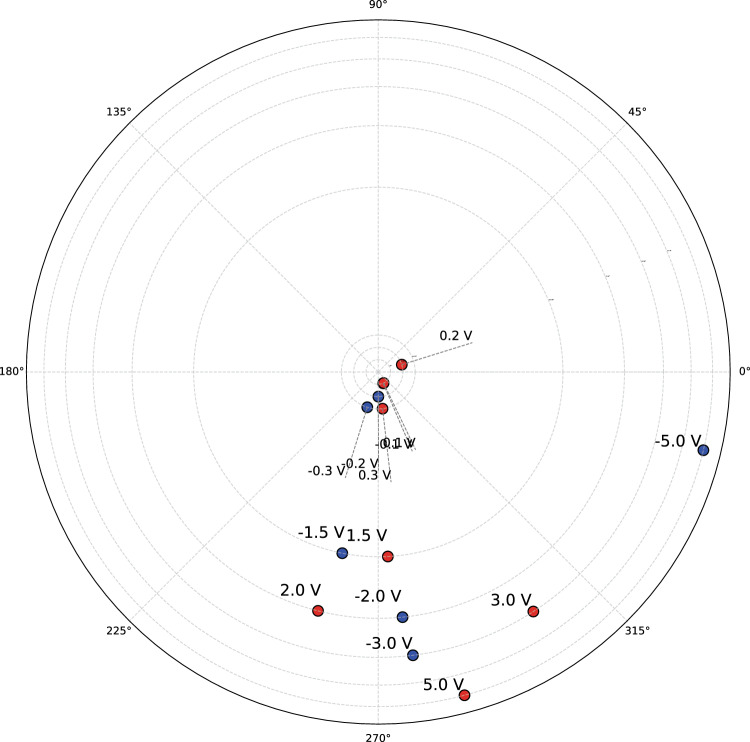
Polar plot of the circular mean values of the phase variation across different applied voltages for the h-DyMnO$_3$ nanocrystal. The red and blue markers represent the circular mean of phase variations for positive and negative voltages respectively. The angular position on the plot corresponds to the circular mean of the phase, while the radial distance represents the voltage. Higher voltages ±3.0 V and ±5.0 V shows increased angular scattering compared to the lower voltages

**Fig. 7:**
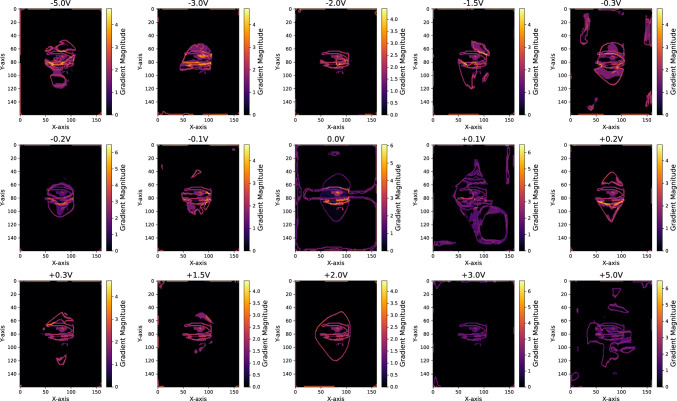
Gradient magnitude of the phase data across different voltages for the h-DyMnO$_3$ nanocrystal. Gradient magnitude plots are shown at each specified voltage, capturing the spatial variation in phase gradient within a central slice of the 3D dataset (XY-plane at the central slice along the Z-axis). The colour scale, presented with the subplots, represents the magnitude of the gradient. The bright region indicates the areas where the domain walls are influenced by the balance between the intrinsic stiffness and the pinning forces caused by impurities or strain within the h-DyMnO3 nanocrystal

In multiferroic materials, the ordered electric dipole moments can be controlled using external electric fields [29, 30]. The dipoles in the material would be expected to line up with the polarity of the applied voltage. Applying a positive voltage orientation aligns the dipoles in one direction, while a negative voltage aligns them in the opposite direction. This realignment process induces changes in the material’s internal structure, which can be detected as phase shifts during the BCDI analysis.

#### Circular mean analysis

As the 3D phase slices provide a visual insight into the phase variations as a function of applied voltages, they do not fully represent the localised phase changes associated with the domain wall structure. Domain walls often correspond to the regions where the crystal phase exhibits significant discontinuity or gradients. For a single Bragg reflection, directly taking the gradient of the phase variation to identify the domain walls may not be an ideal solution, as the phase information can carry an arbitrary offset, leading to incorrect interpretations. To overcome this, the circular mean of the phase variation across voltages was computed, allowing for the assessment of the average phase variation in a manner that accounts for the periodic nature of the phase, providing a more consistent basis for domain wall analysis.[31] The circular mean for a given voltage is calculated by first determining the phase difference relative to a reference voltage (0.0 V in this case). The phase difference, denoted as $\Delta \phi _i = \phi _i - \phi _j$, was computed between each data point at voltage $i$ and the reference phase $\phi _j$, and the circular mean is then given by the following equation:

1 $$\begin{aligned} \text {Circular Mean} = \arctan \left( \frac{\sum \sin (\Delta \phi _i)}{\sum \cos (\Delta \phi _i)} \right) , \end{aligned}$$

where $\Delta \phi _i$ represents the phase difference between each phase measurement and the reference phase. The circular mean effectively represents the average phase orientation, accounting for any phase wrapping. Thus, changes in the circular mean across different voltages may provide insights into domain wall shifts and the evolution of the material’s phase structure under varying electric fields. Table S2 of supplementary file shows the computed circular mean values for each voltage and supplementary figure S11 shows the circular mean histogram.

Figure 6 shows the Polar plot of the circular mean values of the phase variation across different applied voltages for the h-DyMnO$_3$ nanocrystal. The red and blue markers represent the circular mean of phase variations for positive and negative voltages respectively. The radii of the polar plot correspond to the absolute values of the applied voltages on a logarithmic scale, while the angular positions were determined by the circular mean phase values. At lower voltages, the phase angles for the negative and positive voltages are relatively close to each other. The circular mean values of ±0.1 V are overlapping, indicating a low phase variation and a relatively stable phase response of the nanocrystal. As the voltage increases to ±1.5 V, a clear angular deviation was observed in the plot, marking the onset of phase deviation in response to the applied field. At an applied voltage of $-$5.0 V, the circular mean angular position is observed at $-13.5^\circ$, whereas for +5.0 V, the angular position shifts to $-75.1^\circ$. The observed asymmetry between the positive and negative voltages can be attributed to dipole interaction within the h-DyMnO$_3$ nanocrystal. A positive voltage tends to align the dipoles in one direction, while a negative voltage aligns them in the opposite direction.

Figure 7 shows the gradient magnitude of the phase data across different voltages for the h-DyMnO$_3$ nanocrystal. The gradient magnitude plots capture phase variation in the XY-plane at the central slice along the Z-axis, corresponding to the Z-axis slices shown in Figs. 4 and 5. The colour scale, presented with the subplots, represents the magnitude of the gradient. A histogram of the mean gradient magnitude is given in supplementary file Figure S13. At 0.0 V, the gradient magnitude plot shows a distinct bright region with a mean value of 0.0246°/V, indicating areas where the domain walls are influenced by the balance between the intrinsic stiffness and the pinning forces caused by impurities or strain within the h-DyMnO$_3$ nanocrystal.[32, 33] A similar trend is visible in other lower voltages which supports the findings of Fig. 6 where the voltages are insufficient to overcome the pinning forces and induce a phase variation. Since the voltages were scanned from 0.0 V to increasingly negative polarity and then back to positive polarity, there is likely some hysteresis in the response of the dipoles. This hysteresis occurs mainly because the system needs to overcome the ferroelectric coercive field, which requires a non-zero positive voltage. The significant variation observed at +0.1 V aligns with the hysteresis effect, where the domain walls lag behind the change in the applied field, leading to a reorganisation of the domain structure. [34] At higher voltages, particularly at +3.0 V (mean gradient magnitude: 0.0678°/V) and +5.0 V (mean gradient magnitude: 0.0903°/V), the distinct bright region disappears, indicating that the electric field has successfully overcome the pinning forces. As a result, the domain walls were able to move freely and reorganise to minimise the crystal’s internal energy as visible in the gradient magnitude plots.

## Conclusion

In summary, by using 3D Bragg Coherent Diffractive Imaging, we successfully reconstructed a h-DyMnO$_3$ single nanocrystal under applied voltages between ±5.0 V, and demonstrated domain wall pinning due to the intrinsic defects present in the crystal. Our phase gradient magnitude analysis showed that a threshold voltage of +3 V is required to overcome the pinning force, enebling the domain wall motion. These findings provide insights into the interplay between intrinsic defects and domain wall dynamics, contributing to the development of advanced ferroelectric-based devices.

## Methods

### Synthesis

The synthesis of h-DyMnO$_3$ nanocrystals involved a three-step process, beginning with mechanical grinding followed by thermal treatment and a controlled cooling step. The mechanical grinding process was selected due to temporary unavailability of the pulsed laser deposition (PLD) apparatus and its proven efficacy in past experiments. The grinding aimed at producing nanocrystals with an approximate size of 1 micron, suitable for BCDI experiments. A piece of the bulk DyMnO$_3$ material was finely ground in a pestle and mortar, with isopropanol added to facilitate smoother grinding and to prevent agglomeration of the particles.[35, 36] Table S1 of supplementary file shows the specifications of h-DyMnO$_3$ Nanocrystals. The grinding was conducted for a duration of 15 min, ensuring that the resultant powder achieved a uniformly fine consistency without any visible chunks. Following grinding, the powder was divided equally and placed in two ceramic boats. These boats were then inserted into a furnace, one at a time, and heated to 1100 $^\circ$C. The furnace’s temperature was maintained at 1100 $^\circ$C for 60 min to ensure thorough heat treatment. Subsequently, the samples were cooled at two distinct rates: 0.1 $^\circ$C/min and 1 $^\circ$C/min, respectively. This differential cooling was hypothesised to impact the density of ferroelectric vortices in the crystals. Post heat treatment, each powder sample was suspended in isopropanol to facilitate centrifugal separation. This process allowed heavier crystals to settle at the bottom of the vial, leveraging gravity to achieve a rough separation by size. A 10 $\mu$L of the upper layer was then carefully extracted using a micropipette and deposited onto a substrate with Gold interdigitated electrodes (IDEs) printed on them. The IDEs consisted of 5 $\mu m$ wide electrodes and 5 $\mu m$ separation distance. This allowed the deposited crystals to fall in the spaces between the electrodes. The substrate was then spun at 4000 rpm for 10 s using a spin coater, aiming for an even distribution of nanocrystals across the surface, and provide momentum for the crystals placed on top of the electrodes to fall in the spaces between. The size and distribution of the h-DyMnO$_3$ nanocrystals were assessed using an optical microscope. This initial examination confirmed the even distribution of the crystals and provided a preliminary estimate of their size. The synthesis of h-DyMnO$_3$ nanocrystals using mechanical grinding and annealing represents a viable method for producing micron-sized crystals. The mechanical grinding method was successful in synthesizing h-DyMnO$_3$ nanocrystals of the desired size. The experiment utilised a monochromatic beam operating at 9 keV throughout its whole duration. The IDE with 0.1$^\circ$/min cooled crystals was selected for the experiment, and was mounted onto the diffractometer stage. The IDE was connected to a power supply for voltage application across the electrodes.

### Experimental

A code for an area scan was written such that the search for crystals was semi-automated. The code would translate the substrate relative to the beam in perpendicular and parallel directions to illuminate different areas. At each location, a coarse eta scan is performed. Once the area scan is completed, a search through all the data collected is conducted to determine if any potential diffraction patterns could be used. The criteria for choosing a diffraction pattern depend on the centroid’s shape, the intensity of the fringes, and frequency. An isolated diffraction pattern is an essential requirement to avoid any interference. A few crystals were found at the (112) reflection, and one was selected for three-dimensional rocking curve measurements. The crystal was selected based on the fringe frequency, and diffraction pattern response to small voltage application. The last step was necessary to ensure that the crystal was indeed sitting between the electrodes, and not on top of it. A systematic variation in the intensity verifies that the crystal is exposed to the electric field between the electrodes.

A series of electric fields, generated by applying voltages ranging from $-$5.0 V to +5.0 V across the electrodes, were used to induce ferroelectric switching within the material. Three-dimensional rocking curve measurements were taken for each Voltage applied at a long exposure.

## Supplementary information

Supplementary file 1.

## Data Availability

The data underpinning the findings of this study are available from M.C.N upon reasonable request.

## Code availability

The computational analysis in this study utilised “Bonsu: The Interactive Phase Retrieval Suite” software package, and can be downloaded through PyPI (https://pypi.org/project/Bonsu/) or GitHub (https://github.com/bonsudev/bonsu).
