# Supplementary material for: Imaging of electric-field-induced domain structure in DyMnO3 nanocrystals
Source: Discov Nano. 2024 Dec 15;19(1):203. doi: 10.1186/s11671-024-04165-8 (PMC11646962; doi:10.1186/s11671-024-04165-8)
Supplement: Supplementary file 1 [file 11671_2024_4165_MOESM1_ESM.pdf]

# Supplementary File for: Imaging of Electric-Field-Induced Domain Structure in $DyMnO_3$ Nanocrystals

Mansoor A. Najeeb[1*], Robbie Morrison[1], Ahmed H. Mokhtar[1], Daniel G. Porter[2], Frank Lichtenberg[3], Alessandro Bombardi[2], Marcus C. Newton[1*]

[1]Department of Physics & Astronomy, University of Southampton, United Kingdom.

[2]Diamond Light Source, Harwell Oxford Campus, Didcot, United Kingdom.

[3]Department of Materials, ETH Zürich, Zürich, Switzerland.

*Corresponding author(s). E-mail(s): m.nellikkal@soton.ac.uk; m.c.newton@soton.ac.uk;

**Table S1** Specifications of $DyMnO_3$ Nanocrystals

| Property | Value |
|---|---|
| **Lattice (Conventional)** | |
| a | 6.14 Å |
| b | 6.14 Å |
| c | 11.43 Å |
| **Symmetry** | |
| Crystal System | Hexagonal |
| Lattice System | Hexagonal |
| Hall Number | P 6c -2 |
| International Number | 185 |
| Symbol | $P6_3cm$ |
| Point Group | 6mm |
| **Lattice Parameters** | |
| $\alpha$ | 90.00 º |
| $\beta$ | 90.00 º |
| $\gamma$ | 120.00 º |
| **Volume** | 372.65 $Å^3$ |

**Table S2** Circular Mean Values for Different Applied Voltages

| Voltage (V) | Circular Mean (radians) |
|---|---|
| -5.0V | -0.236 |
| -3.0V | -1.449 |
| -2.0V | -1.472 |
| -1.5V | -1.767 |
| -0.3V | -1.873 |
| -0.2V | -1.568 |
| -0.1V | -1.165 |
| 0.0V | 0.000 |
| +0.1V | -1.123 |
| +0.2V | 0.302 |
| +0.3V | -1.455 |
| +1.5V | -1.519 |
| +2.0V | -1.818 |
| +3.0V | -0.996 |
| +5.0V | -1.310 |

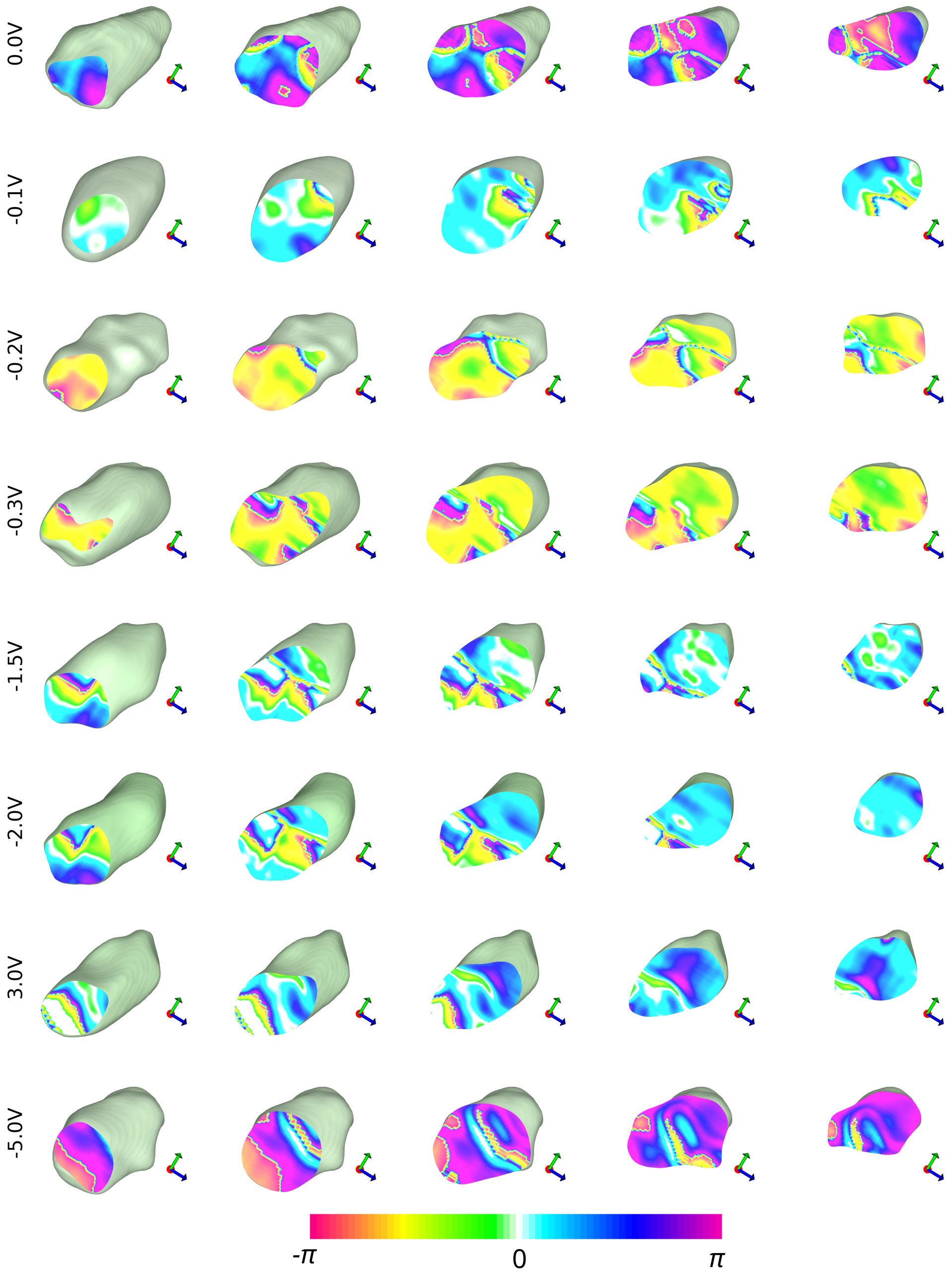


**Fig. S1** Slices of reconstructed h-$DyMnO_3$ nanocrystal under negative voltages sliced along x-axis.

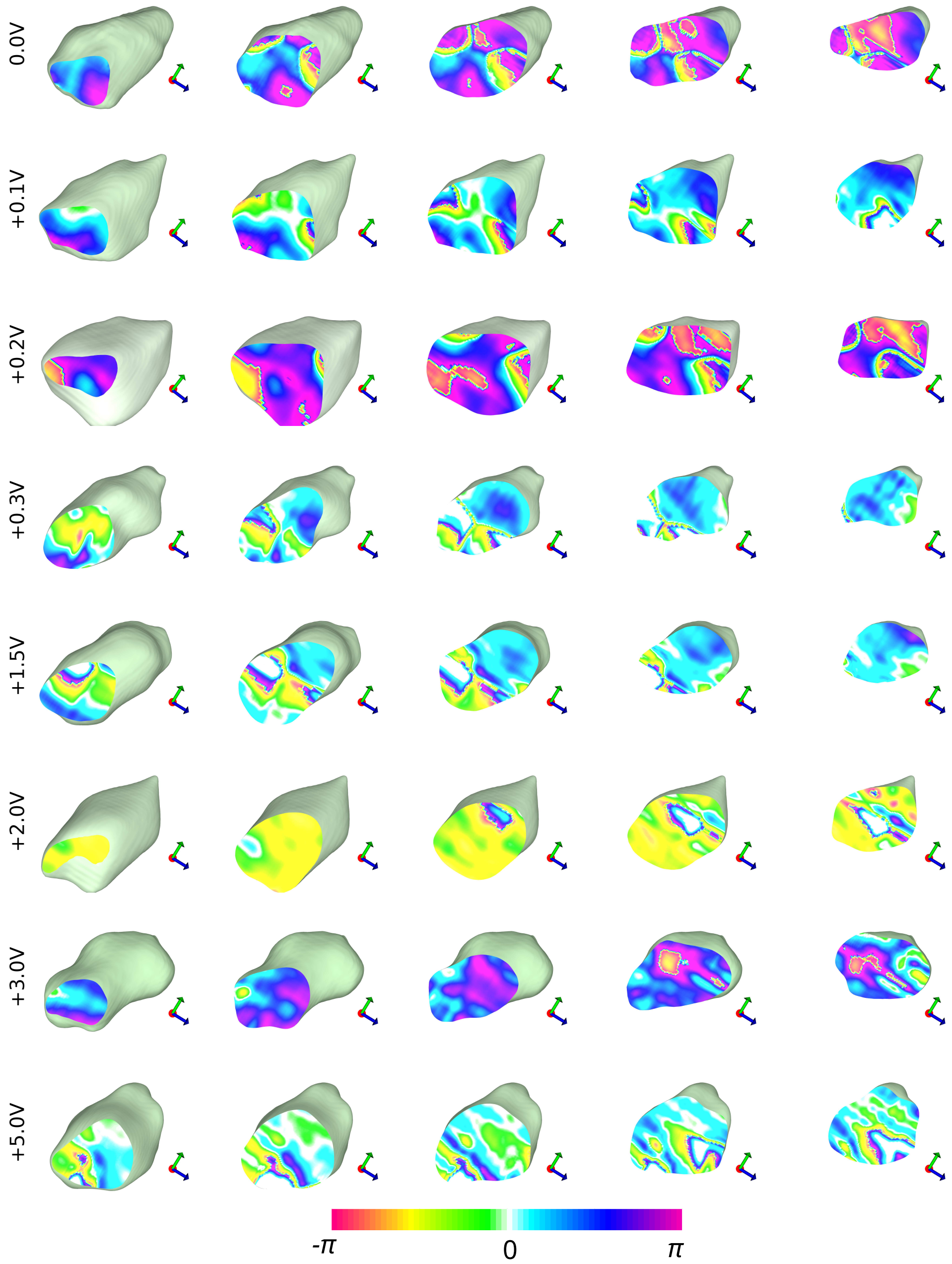


**Fig. S2** Slices of reconstructed h-$DyMnO_3$ nanocrystal under positive voltages sliced along x-axis.

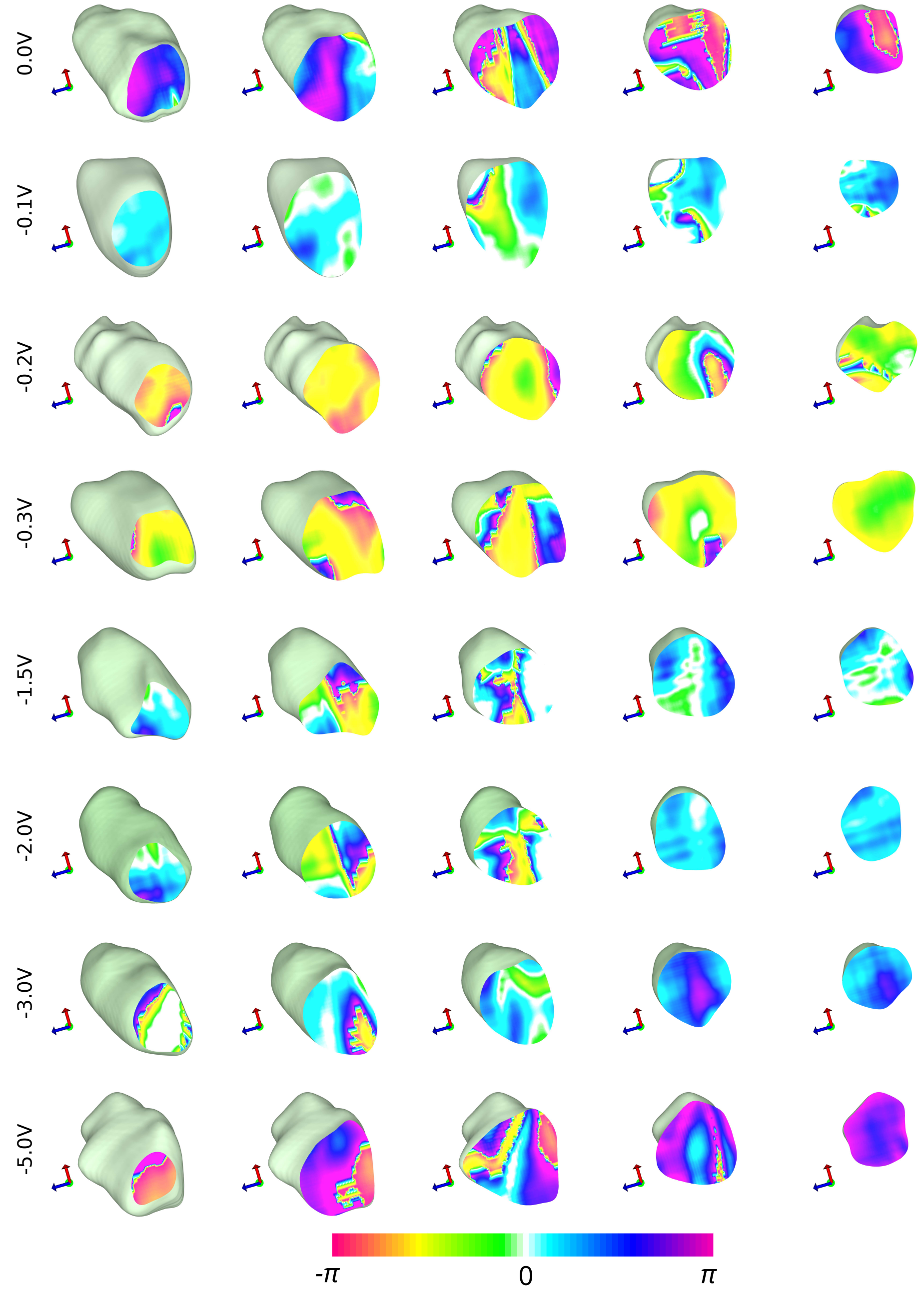

**Fig. S3** Slices of reconstructed h-$DyMnO_3$ nanocrystal under negative voltages sliced along y-axis.

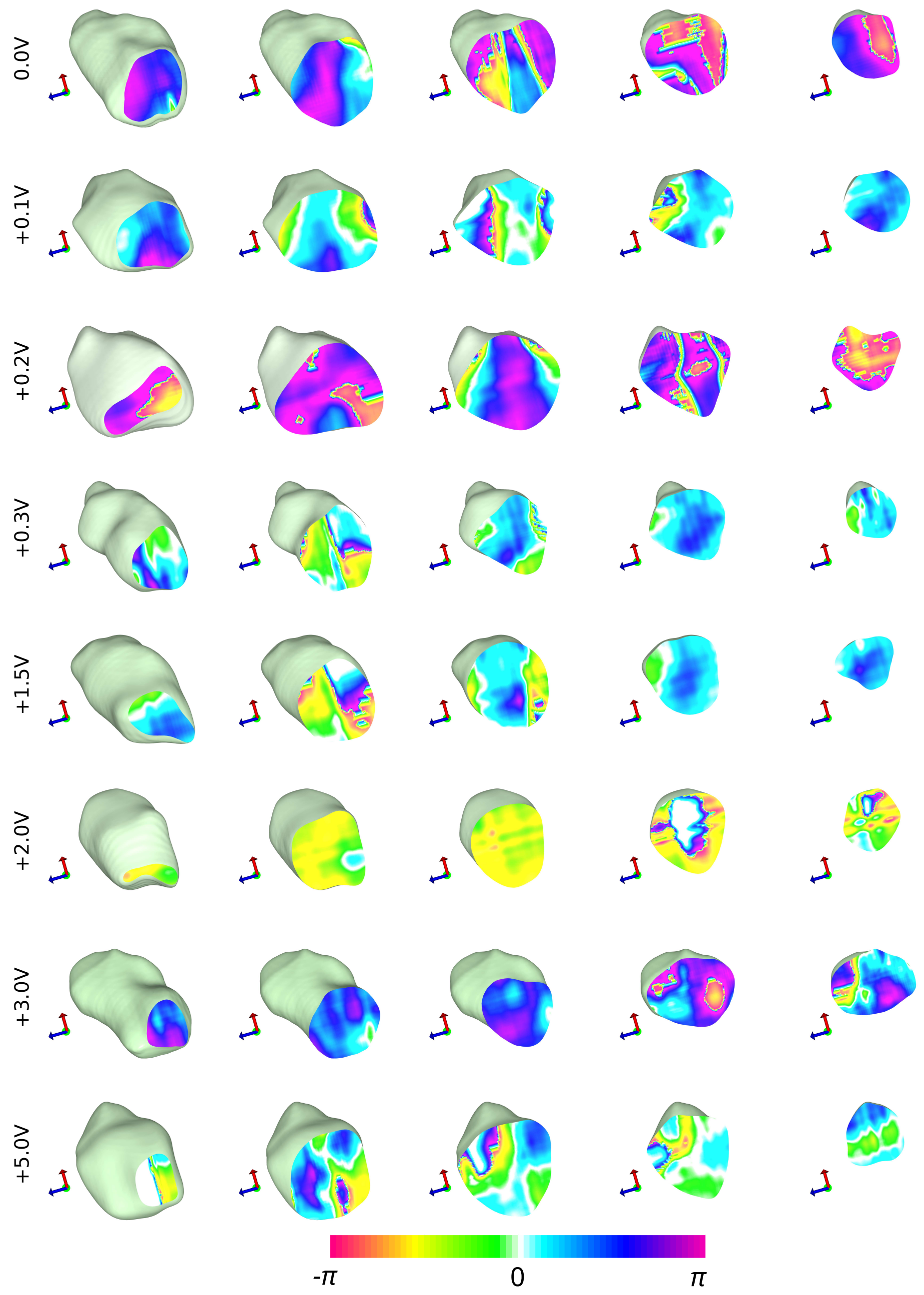

**Fig. S4** Slices of reconstructed h-$DyMnO_3$ nanocrystal under positive voltages sliced along y-axis.

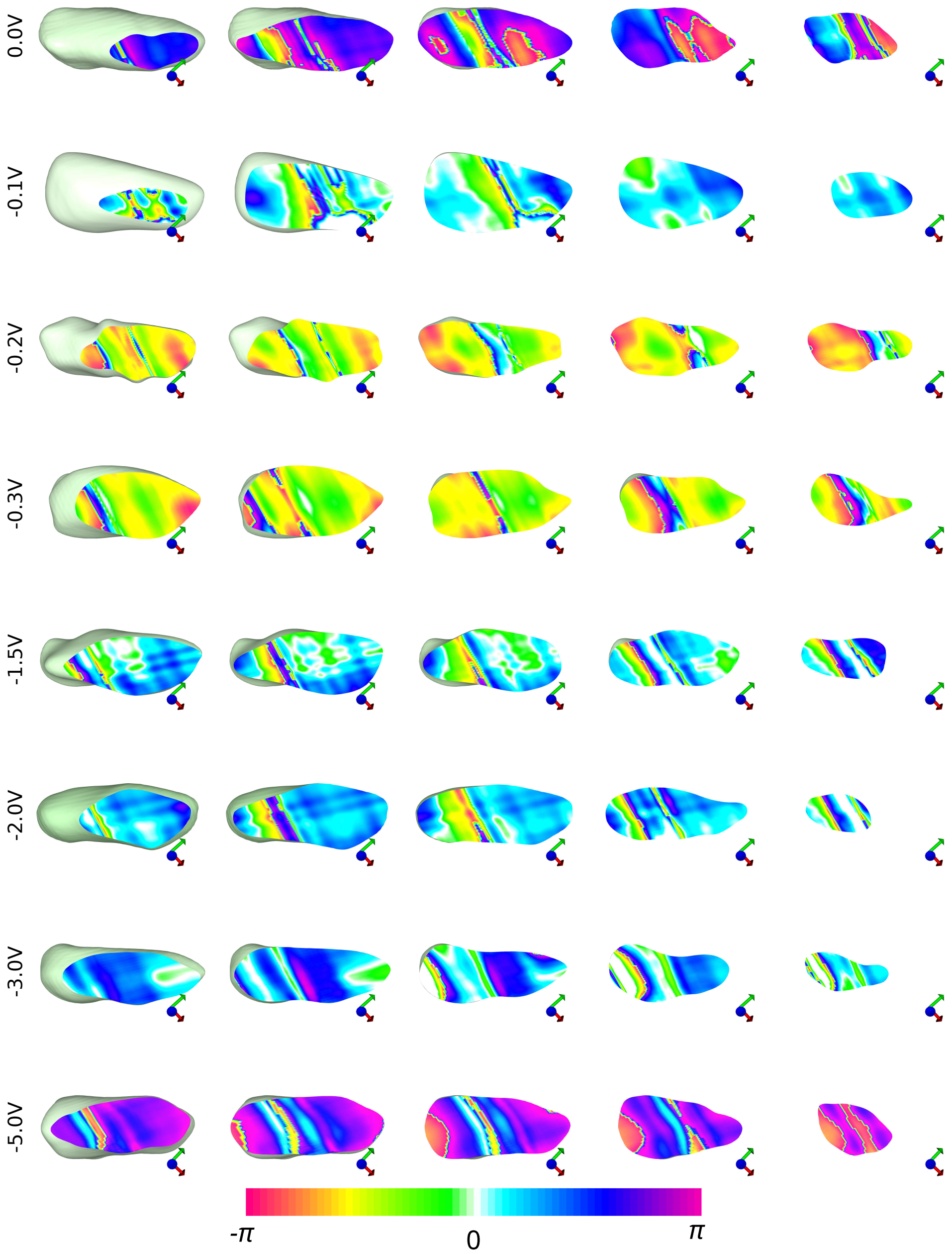


**Fig. S5** Slices of reconstructed h-$DyMnO_3$ nanocrystal under negative voltages sliced along z-axis.

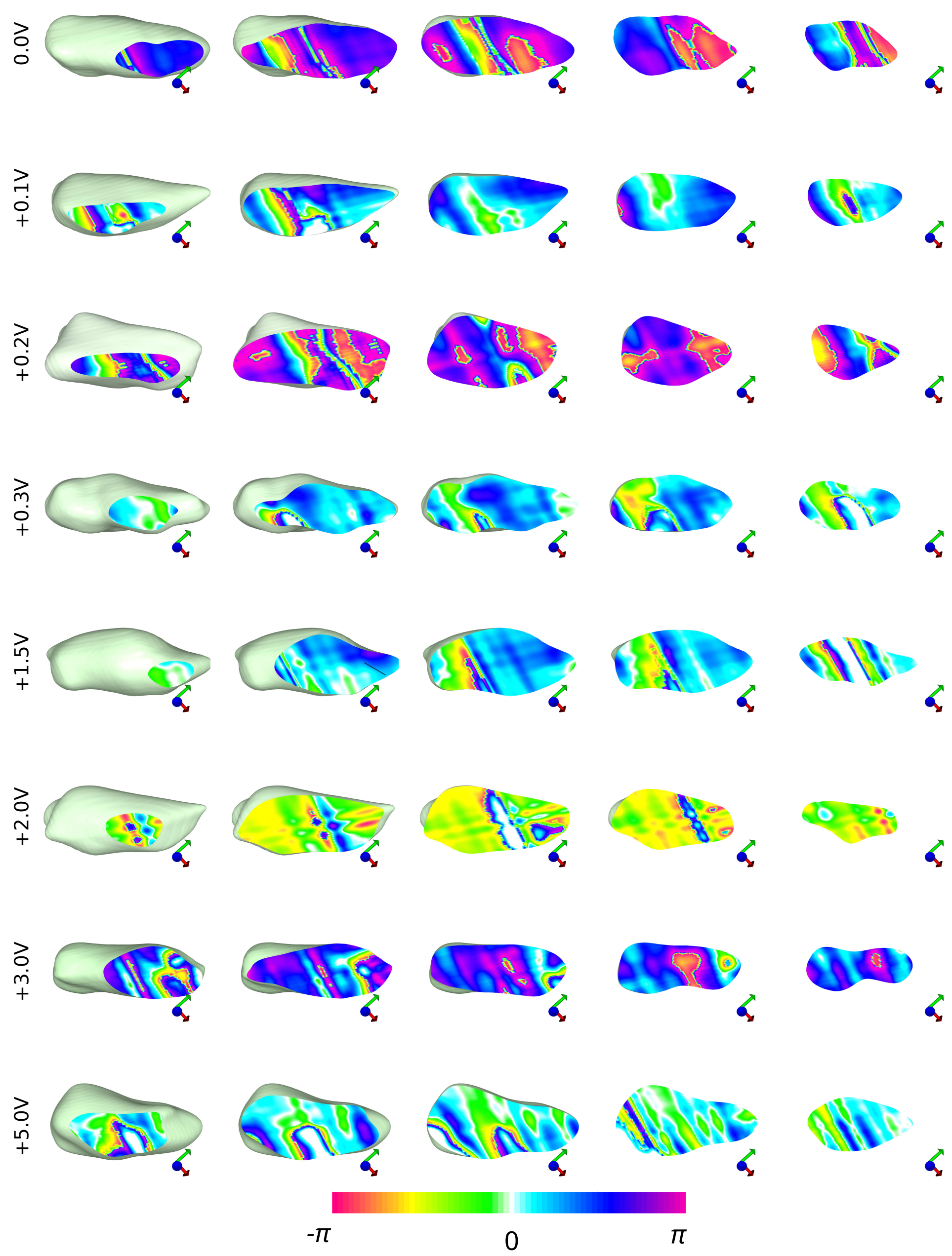


**Fig. S6** Slices of reconstructed h-$DyMnO_3$ nanocrystal under positive voltages sliced along z-axis.

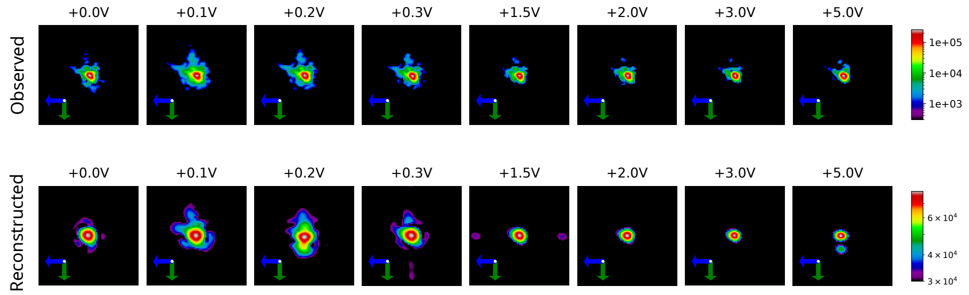


**Fig. S7** Comparison of observed and reconstructed diffraction pattern of positive voltages along x-axis.

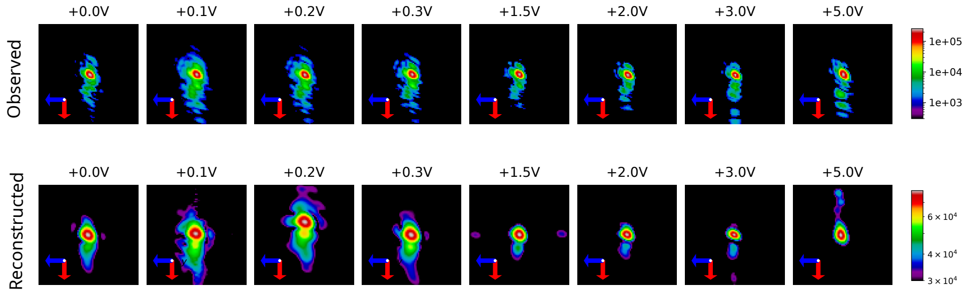


**Fig. S8** Comparison of observed and reconstructed diffraction pattern of positive voltages along y-axis.

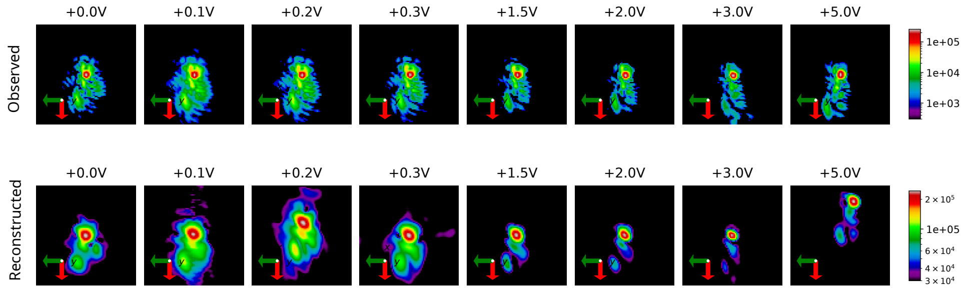


**Fig. S9** Comparison of observed and reconstructed diffraction pattern of positive voltages along z-axis.

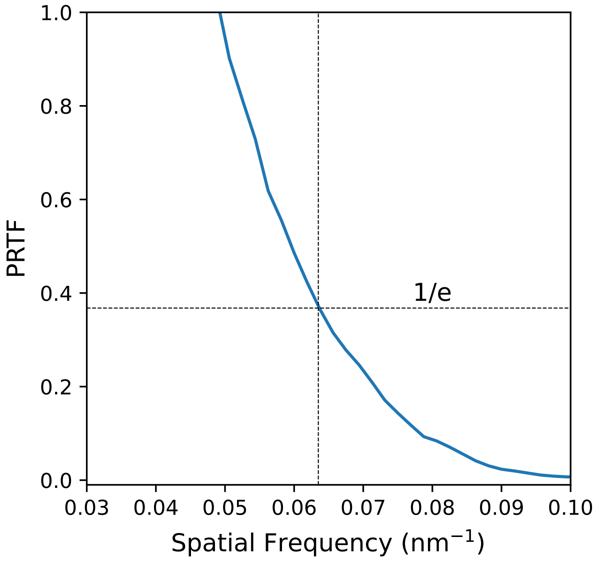


**Fig. S10** Phase Retrieval Transfer Function (PRTF) used to estimate the resolution of 15.83 nm. Resolution is determined at the spatial frequency where the PRTF curve drops to $\frac{1}{e}$ .

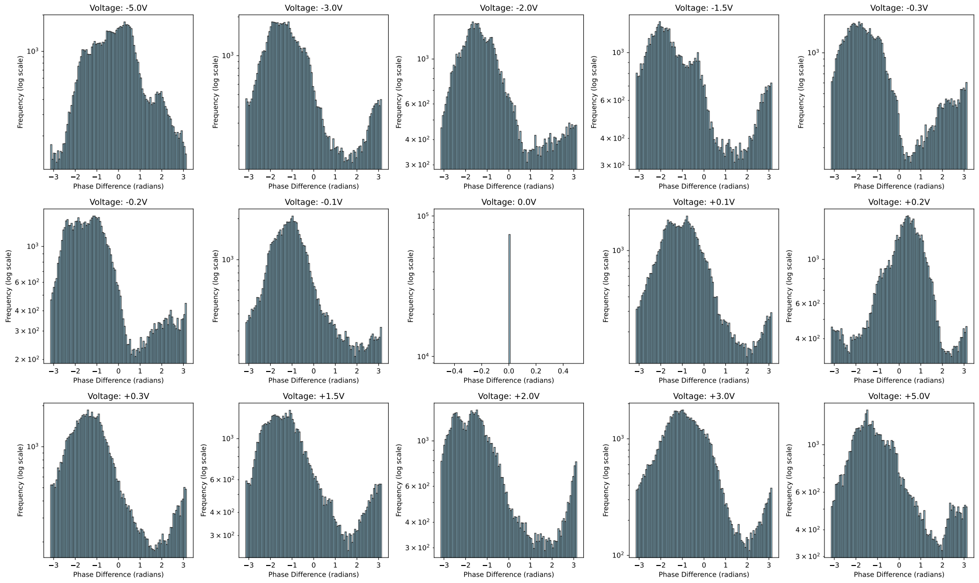


**Fig. S11** Circular mean phase histograms of the reconstructed h-$DyMnO_3$ nanocrystal at various applied voltages, showing the distribution of phase difference (in radians) from $-\pi$ to $\pi$.

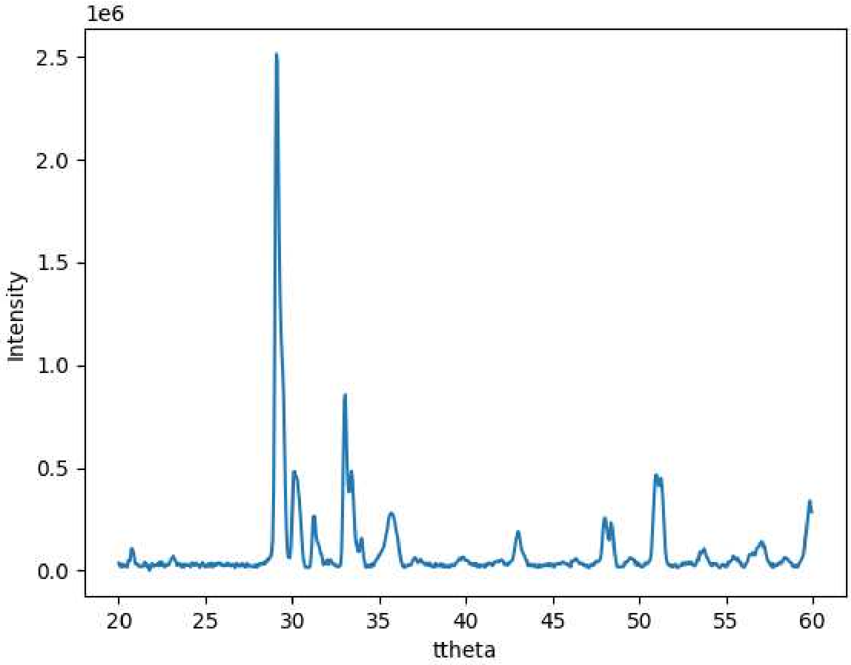


**Fig. S12** XRD scan of $DyMnO_3$ nanocrystals after heat treatment at 1100 °C and gradually cooled at 0.1 °C/min.

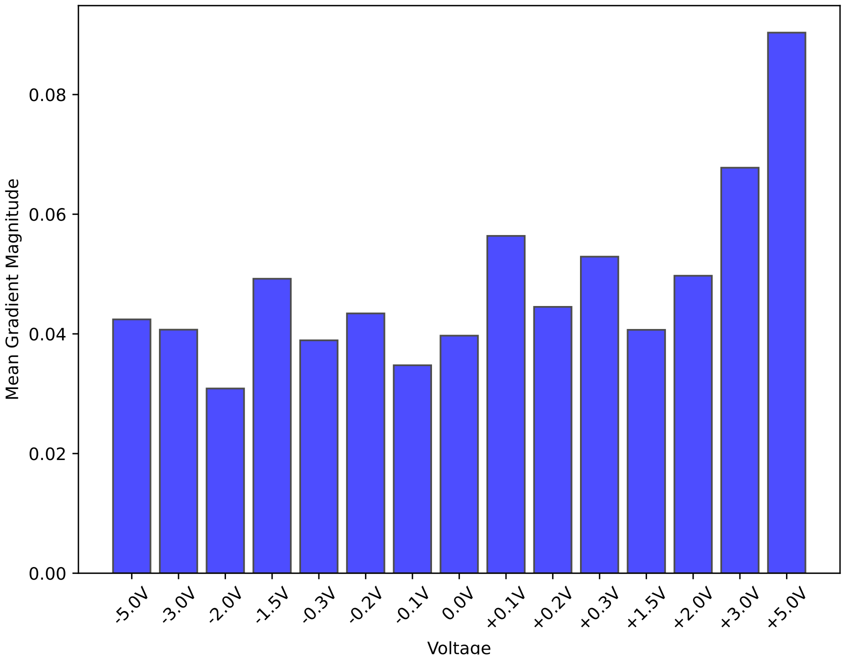


**Fig. S13** Mean gradient histogram at different applied voltages.
